# Management of cerebral abscess with large ventricular septal defect in rural area: a case report

**DOI:** 10.1093/jscr/rjad703

**Published:** 2023-12-30

**Authors:** Donny Argie, Patrick Putra Lukito, Sugi Deny Pranoto Soegianto, Leonora Johana Tiluata, Christopher Lauren

**Affiliations:** Department of Surgery sub Neurosurgery, Faculty of Medicine, RSUD WZ Johannes Kupang, Universitas Nusa Cendana, East Nusa Tenggara 85111, Indonesia; Department of Surgery sub Neurosurgery, Faculty of Medicine, RSUD WZ Johannes Kupang, Universitas Nusa Cendana, East Nusa Tenggara 85111, Indonesia; Department of Paediatrics, Faculty of Medicine, RSUD WZ Johannes Kupang, Universitas Nusa Cendana, East Nusa Tenggara 85111, Indonesia; Department of Cardiology, Faculty of Medicine, RSUD WZ Johannes Kupang, Universitas Nusa Cendana, East Nusa Tenggara 85111, Indonesia; Neurosurgery Division, Department of Surgery, Faculty of Medicine, Universitas Udayana, Prof. Dr. I.G.N.G. Ngoerah General Hospital, Denpasar, Bali 80113, Indonesia

**Keywords:** cerebral abscess, neuroinfection, neurosurgery, ventricular septal defect

## Abstract

Cerebral abscess is an uncommon complication of cyanotic heart disease. However, it has a high case fatality rate, and its management requires a multidisciplinary approach. Earlier diagnosis would result in a better outcome. In this report, we presented a case of a 6-year-old boy with a cerebral abscess and a large ventricular septal defect, which was treated surgically in a rural area with a limited resources facility.

## Introduction

Severe congenital heart disease disrupts normal blood flow through the heart and lungs, causing a cyanotic condition where there is oxygen depletion in the body. Cerebral abscess is a complication that may arise from cyanotic congenital heart disease [1]. This condition is a challenging clinical problem with substantial case fatality rates [2]. Managing cerebral abscess requires an immediate multidisciplinary approach, including surgical and nonsurgical treatments. We report a case of a 6-year-old diagnosed with a ventricular septal defect (VSD) and cerebral abscess. Due to facility limitations, this patient was diagnosed late and was untreated for years. This longstanding condition led to the occurrence of a bidirectional shunt that resulted in a cyanotic condition and eventually led to a complication in the form of a cerebral abscess.

## Case report

A 6-year-old boy came to the emergency department with a chief complaint of decreased consciousness 1 week ago. The patient also experienced a fever 2 weeks ago. The patient was hospitalized for 18 days in the district hospital before being referred to our health center. During the hospitalization in the district hospital, the patient experienced seizures two times and began having a decreased consciousness ever since. The patient had a history of cyanosis since the age of 4 months. The cyanosis persisted even when the patient was resting. However, the patient’s family did not seek medical treatment.

**Figure 1:**
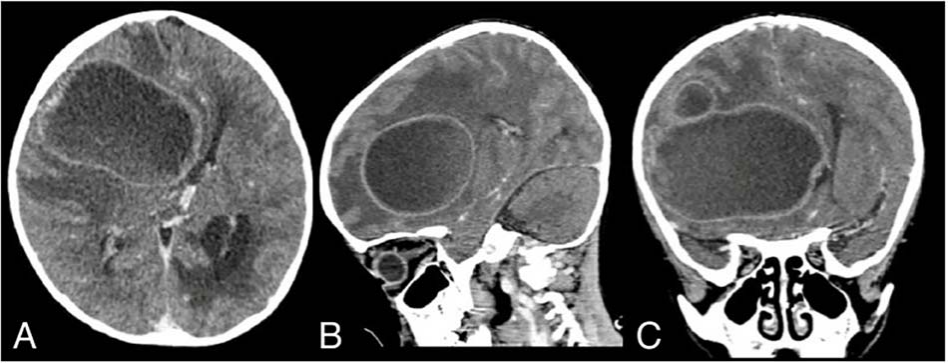
Admission contrast head CT scan showing a large hypodense lesion with ring-enhancement on the right frontotemporoparietal lobe; (A) axial view; (B) sagittal view; (C) coronal view.

On admission, his GCS was E2M4V2 with a blood pressure of 88/58 mmHg, heart rate of 94 times per minute, respiratory rate of 33 times per minute, temperature of 36.6°C, and oxygen saturation of 86%. Auscultation revealed a systolic murmur. The patient also had cyanosis and clubbing fingers. The patient was malnourished, with a weight of 13 kg and a height of 98 cm. His chest X-ray revealed a cardiomegaly. Head CT scan revealed a hypodense lesion on the right frontotemporoparietal lobe with a size of 5.7 × 6.5 × 7.7 cm with tentacle-like edema on its surrounding, which gave a ring-enhancement after contrast administration (Fig. 1A–C). On echocardiography, there was a significant VSD with a bidirectional shunting.

The patient was then diagnosed with cerebral abscess and congenital VSD and was admitted to the intensive care unit. The patient then received Ceftriaxone IV 2 × 550 mg, Metronidazole IV 3 × 110 mg, Dexamethasone IV 3 × 2.5 mg, Furosemide IV 2 × 10 mg, Omeprazole IV 2 × 10 mg, and Ondansetron IV 3 × 2.5 mg. The patient initially improved, and he regained full consciousness during the first day of admission. However, on the next day, the patient’s condition rapidly deteriorated, and the patient’s GCS decreased to E1M5V1. An emergency evacuation craniotomy was performed, and the abscess was drained. Cytological examination of the abscess capsule and pus supported the diagnosis of a cerebral abscess. However, the microbiological culture of the pus returned negative, which might have been caused by the administration of antibiotics before the surgery.

**Figure 2:**
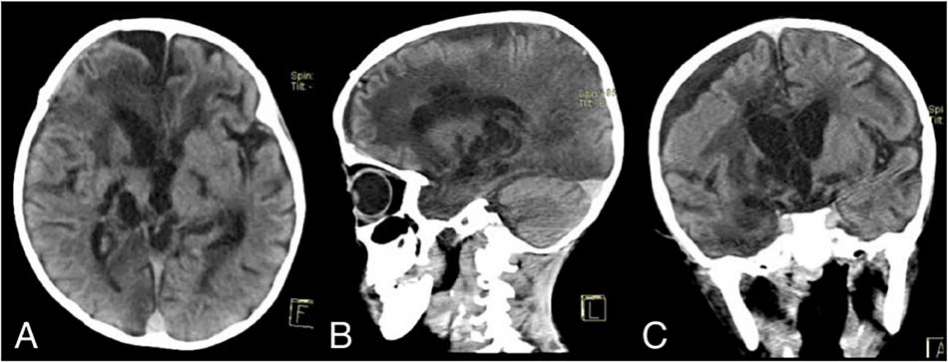
Discharge noncontrast head CT scan showing that the abscess has been completely removed; (A) axial view; (B) sagittal view; (C) coronal view.

On Postoperative Day 2, the patient’s consciousness improved with GCS E2M5V2. The patient was given Ceftriaxone IV 2 × 750 mg, Metronidazole IV 3 × 110 mg, Paracetamol IV 4 × 150 mg, Captopril PO 3 × 3.125 mg, Furosemide PO 2 × 5 mg, and Digoxin 2 × 0.0625 mg. The patient was transferred to the nonintensive care ward on Postoperative Day 18. During hospitalization, the patient continued to have a fever, and he also contracted pneumonia. His sputum culture isolated *Pseudomonas aeruginosa* with resistance to Ampicillin, Cefepime, Ceftazidime, Meropenem, Ciprofloxacin, and Cotrimoxazole. Fortunately, the pathogen was still sensitive to Amikacin, and the patient was promptly given Amikacin IV 1 × 200 mg. The patient’s condition continued to improve, and after being 1 week free of fever, the patient was discharged after 50 days of hospitalization. His GCS on discharge was E3M5V2, with oxygen saturation of 70–80% without supplemental oxygen. A noncontrast head CT scan on discharge showed that the abscess had been completely removed (Fig. 2A–C).

## Discussion

A cerebral abscess is a collection of pus localized by a well-vascularized capsule due to bacterial invasion of the brain [1]. The most commonly identified pathogens in patients with cerebral abscesses are *Streptococcus* spp., *Staphylococcus* spp., and gram-negative bacteria. Intracerebral bacterial invasion may originate from contiguous spread or hematogenous spread [2]. Cyanotic heart disease occurs when the blood shunts from the right to the left side of the heart, resulting in nonfiltered blood in the bloodstream. The most common form was Tetralogy of Fallot, followed by VSD and transposition of great vessels [3].

For patients with cyanotic congenital heart defects, bacteria may enter the cerebral circulation. Other predisposing factors in these patients are severe hypoxemia and compensatory polycythemia that result in minute-low perfusion areas, metabolic acidosis, and increased blood viscosity. Altogether, these conditions lead to a focal area prone to infection, and when seeded by the shunted blood containing microorganisms, it will form a cerebral abscess [4]. Cerebral abscess is an uncommon complication of cyanotic congenital heart disease; a previous study found the incidence of cerebral abscess in patients with congenital heart disease was 18% [1].

Management of cerebral abscesses requires a multidisciplinary approach and aims to reduce space-occupying activity, reduce intracranial pressure, and eradicate pathogenic organisms. Surgical management includes aspiration and excision. For small and deep lesions, surgical aspiration is the recommended treatment. With the presence of multiple and larger lesions or midline shifts, surgical excision is recommended [5, 6]. The head CT scan of our patient revealed a large lesion with subsequent hydrocephalus and midline shift. Together with the progressive neurologic deficit of our patient, these were the indications for emergency surgery.

Conservative treatment includes the administration of antibiotics. Broad-spectrum antibiotics should be administered as an initial therapy before the culture results are available. Cefotaxime/Ceftriaxone/Ceftazidime, Vancomycin, and Metronidazole are some empiric antibiotics that are commonly used [1, 7]. Antibiotic treatment is typically administered for 4–8 weeks, depending on the clinical and radiographic resolutions [6].

In our case, the patient was diagnosed with VSD at the age of 6 years old, which was late. Irreversible pulmonary veno-occlusive disease had likely developed. However, with all the current limitations, we have successfully operated and prevented further neurological deficits or even death. We have shown that cerebral abscess can be managed successfully in rural areas, and with earlier treatment where the patient’s neurological deficits were still mild, a better recovery is possible.
